# Melatonin/P34HB Films for Active Packaging: Optimizing Flavor Preservation and Quality of Honey Peaches During Storage

**DOI:** 10.3390/foods14050869

**Published:** 2025-03-03

**Authors:** Sunxiao Tantai, Jiayi Xu, Wenya Ma, Xiaofang Liu, Li Li, Yifen Wang

**Affiliations:** 1 College of Food Science and Technology, Shanghai Ocean University, Shanghai 201306, China; 2 Engineering Research Center of Food Thermal-Processing Technology, Shanghai 201306, China; 3 Biosystems Engineering Department, Auburn University, Auburn, AL 36849-5417, USA

**Keywords:** melatonin, P34HB, honey peach, flavor, food packaging

## Abstract

To address unpredictable flavor changes in postharvest peaches during storage, this study investigated the use of bioactive packaging with melatonin-infused P34HB films. Films with melatonin concentrations of 0%, 1%, 3%, and 5% were prepared using the extrusion casting method and applied to peach storage at room temperature. Comprehensive film properties were characterized, showing that melatonin minimally impacted the films’ mechanical properties, including gas and water vapor permeability, but significantly increased film haze. Using GC-IMS, 30 organic compounds affecting peach flavor were effectively identified, including 8 aldehydes, 5 alcohols, 4 ketones, 12 esters, 1 pyrazine, 1 olefin, and 1 furan. Unpackaged, naturally ripening peaches served as a reference for assessing flavor and quality changes across various packaging groups during storage. The results indicated that the appearance of off-flavor organic compounds, such as ethanol produced by peach anaerobic respiration and complex esters, was the primary cause of flavor deterioration. The P34HB film with 1% melatonin most effectively preserved the original flavor and juiciness of peaches, highlighting its potential as an active packaging solution for fruit.

## 1. Introduction

Peaches are highly favored by consumers for their soft, juicy texture and distinct flavor profile [1,2]. However, their high respiration rate and delicate, easily damaged skin make them prone to spoilage during postharvest storage and transport [3], which considerably diminishes their commercial value [4].

Traditional peach preservation techniques, such as modified atmosphere packaging [5]; natural preservatives, like essential oil [6,7]; and chemical preservatives, including nanoparticles [8,9], have shown some success in extending shelf life. However, few studies have explored packaging suitability specifically for flavor retention. Therefore, developing a new packaging technology that can both extend shelf life and maintain the flavor and quality of peaches addresses a significant need in the market.

Melatonin (MT), a multifunctional small molecule found in both plants and animals, is known to regulate plant growth and enhance stress tolerance [10]. Studies have shown that melatonin can effectively extend fruit shelf life by inhibiting microbial growth, mitigating oxidative stress, and delaying senescence [11]. The traditional application of melatonin involves immersion treatment of peaches [12], a complex method requiring technical expertise and equipment. This approach does not consider packaging needs for extended storage and transportation. Recent research has shifted focus toward incorporating melatonin into packaging to extend the shelf life of fruits and vegetables [13].

Biodegradable polymers are increasingly used as food packaging materials to mitigate environmental impacts [14]. Among these, poly3-hydroxybutyrate4-hydroxybutyrate (P34HB) film offers high mechanical strength, excellent barrier properties, and biodegradability [15,16]. Traditionally, P34HB films have been produced using solvent casting [17,18] and hot-press methods [19,20], which are less suitable for mass production. Extrusion casting provides a more efficient approach to meet market needs. While PHB films with antimicrobial agents have been applied for food preservation [21,22], concerns around the safety and impact of these agents on food quality, particularly in terms of odor, remain key considerations for packaging applications.

In this study, P34HB film with varying concentrations of melatonin were prepared by extrusion casting to develop a novel active packaging solution. The morphology, mechanical properties, and optical and barrier properties of these new composite films were determined comprehensively. Peaches were used as the test subjects to examine the effects of P34HB films with different melatonin concentrations on basic physical and chemical properties, as well as flavor changes during postharvest storage at room temperature. Objective analysis using GC-IMS, paired with subjective sensory evaluation, was employed to identify the most suitable packaging material for preserving peach flavor.

## 2. Materials and Methods

### 2.1. Materials

Melatonin and tributyrin were purchased from Maclean’s (Shanghai, China) P34HB was purchased from Beijing Micro Structure Workshop Biotechnology Co., Ltd. The peaches were purchased from Shanghai Miao yang Fruit and Vegetable Professional Cooperative in July 2024.

### 2.2. Preparation of MT/P34HB Active Packaging Film

The MT/P34HB films were prepared using an extrusion casting method based on Zhao et al. [23]. Before granulation, P34HB powder was baked at 80 °C for 8 h to remove moisture. A high-speed shear mixer was then employed to ensure uniform blending, mixing P34HB with 10% plasticizer (Tributyrin) and varying proportions of melatonin. The mixture was pelleted with a granulator and then processed into film using a twin-screw extruder (Ke chuang Equipment Co., Ltd., Shanghai, China). The extruder heating zones were set to gradually increase in temperature from 140 °C to 170 °C, with a screw speed of 60 rpm. The winder speed was set to 3 rpm, and the thickness of the film was about 40 μm. The different film compositions, based on melatonin concentration, were designated as follows: (A) P34HB control, (B) 1% MT/P34HB, (C) 3% MT/P34HB, and (D) 5% MT/P34HB, where melatonin content is expressed as a percentage of the total substrate weight (plasticizer and P34HB).

### 2.3. Characterization of Films

#### 2.3.1. Fourier-Transform Infrared Spectroscopy (FTIR)

Four types of MT/P34HB films with different melatonin concentrations were characterized by Nicolet iS10 Fourier-Infrared Spectrometer (Thermofisher Scientific, Waltham, MA, USA) to assess the impact of melatonin incorporation on the chemical composition and structure of P34HB. The spectral resolution was set to 4 cm^−1^, with 32 scans conducted for each sample to ensure high data resolution and a high signal-to-noise ratio.

#### 2.3.2. X-Ray Diffraction (XRD)

The crystal structure of the films was characterized using Ultima IV polycrystalline powder diffractometer (Rigaku Corporation, Tokyo, Japan), as described by [24]. XRD patterns were obtained using Cu-Kα radiation at a tube voltage of 40 kV and a tube current of 40 mA, with a scanning angle (2θ) ranging from 5° to 80° and a scanning rate of 5°/min.

#### 2.3.3. Scanning Electron Microscopy (SEM)

The surface and cross-sectional morphology of the films were measured with a scanning electron microscope (Hitachi SU5000, Tokyo, Japan). Small samples were taken from each of the four different types of films for surface morphology analysis to obtain the cross-sectional structural information. All film samples were quickly quenched in liquid nitrogen. The samples were then gold-plated for 60 s, and the surface and cross-section images were captured at an acceleration voltage of 10 kV.

#### 2.3.4. Comprehensive Properties of Films

##### Physical Properties

The mechanical properties of packaging films, including tensile strength (TS, MPa) and elongation at break (EAB, %), were evaluated according to the method of Zhao et al [23]. Film samples were cut into 100 mm × 15 mm for test using an XLW(EC) intelligent electronic tensile tester (Labthink Instruments Co., Ltd., Jinan, China). Test conditions included a stretching rate of 50 mm/min and an initial clamping distance of 50 mm.

##### Optical Properties

For the optical properties, the transmission (T, %) and haze (H, %) of the film were measured using a WGT-S light transmittance (Fog meter, Shanghai, China), and the same sample was measured three times at different points.

##### Barrier Properties

The barrier properties of the films were evaluated using oxygen transmission rate (OTR), water vapor transmission rate (WVTR), and water contact angle (WCA) as key indicators.

The OTR was measured according to the method of Jiang et al. [25]. The film samples were securely placed in the detection chamber of an i-OXTRA 7700 gas penetration tester (Labthink, China) at 23 °C and 50% relative humidity to ensure system integrity for precise OTR measurement.

The WVTR of the film was measured using a W-B-31E WVTR tester (Lab Stone, Co., Ltd., Guangzhou, China) in accordance with the the method of Jiang et al. [25] at 38 °C and 10% relative humidity, using the weight-reduction method.

The WCA of the film was measured with an SDC-100 contact angle-measuring instrument (Dongguan, China). A 4 μL droplet of deionized water was applied to the film surface, and an image of the membrane surface was captured to WCA.

### 2.4. Peach Sample Treatment

A specific cultivar of peach fruit, known as ‘Nan hui Honey Peach’, was selected for this study. Peaches were collected from an orchard located in Pudong New Area of Shanghai, China in July 2024 (geographical coordinates: 30°93’ N, 121°83′ E). To ensure quality, all peaches were promptly transported to the laboratory within 6 h after harvesting. In the laboratory, peaches with uniform weight and ripeness and no mechanical damage were chosen as experimental samples.

The experimental design comprised a control group and four experimental groups, each group consisting of three parallel samples, with each individually packaged. The average weight of each sample was precisely controlled at 198.5 ± 8.3 g. The samples were sealed using a Model SF-200 heat sealing machine (Tianlu Machinery Equipment Co., Ltd., Jinan, China) in bags measuring 150 mm × 150 mm. The samples were randomly divided into five groups; four groups were packaged in P34HB bags containing varying concentrations of melatonin, while the remaining group served as a control and remained unpackaged. All samples were stored at room temperature (24 °C). Physicochemical changes in each sample group were analyzed at specified intervals: days 0, 2, 4, 6, and 8.

#### 2.4.1. Basic Physicochemical Indexes of Peach

The basic physicochemical indexes of peaches were evaluated by the weight loss rate (*WLR*), firmness, and soluble solids content.

The *WLR* was measured using the weighing method. The initial mass of the sample was recorded, followed by periodic measurements of its mass.

(1) $$\mathrm{WLR}\;\left(\%\right)=\frac{\left(W_{0}-W_{1}\right)}{W_{0}}\times \;100\%$$

Peach firmness was measured using a handheld hardness tester. The probe was inserted vertically into the peach pulp, and the hardness values were recorded in Newtons (N). To ensure data accuracy, measurements were taken at the shoulder of each fruit, with multiple readings obtained and averaged.

Soluble solids content was determined using a handheld digital refractometer. A small sample of peach, taken approximately 1 cm below the skin at the equatorial region of the peach, was ground to extract juice. A drop of juice was then placed onto the detection area of the refractometer, and the soluble solids content was recorded in percentage (%).

#### 2.4.2. Headspace Composition Analysis

A test needle was inserted into the bag to record the O_2_ and CO_2_ content using the Check Mate 9900 headspace analyzer (Shanghai, China).

#### 2.4.3. Flavor Substances Analysis

Volatile compounds were analyzed using the FlavourSpec^®^ Gas Chromatograph–Ion Mobility Spectrometer (GC-IMS) flavor analyzer (G.A.S., Dortmund, Germany). A 2.0 g fruit sample was placed in a 20 mL headspace glass vial and incubated at 40 °C for 15 min. After incubation, 300 μL of the headspace phase was automatically injected into the injector at 45 °C in unshelled injection mode, using a syringe. Volatile components were separated by gas chromatography (GC) with an MXT-WAX column (30 m × 0.53 mm, 1.0 μm) coupled to IMS at 45 °C. High-purity nitrogen (99.999%) was used as the carrier gas with the following flow conditions: initially maintained at 2 mL/min for 2 min, and then linearly increased to 100 mL/min over 10 min, before stopping the flow. In the IMS ionization chamber at 45 °C, analytes were ionized, and drift gas (N_2_) was set at a flow rate of 150 mL/min. All analyses were performed in triplicate. Volatile compounds were identified by comparing drift times with retention indices (RIs) and using the GC-IMS libraries, as well as standards from the NIST 2020 database.

#### 2.4.4. Sensory Evaluation Method

Samples were photographed throughout storage. Slightly modified according to the method of [26], sensory evaluation was conducted by eight trained professionals from the College of Food Science, Shanghai Ocean University, who assessed peach quality based on sensory standards listed in Supplementary Table S1. Scoring was performed on a percentage basis, with the following categories: excellent, total score ≥ 85; good, 70 points ≤ total score < 85 points; general, 55 points ≤ total score < 70 points; and poor, total score < 55.

### 2.5. Data Processing

A range of tools and methods were employed in this research for data analysis. Firstly, single-factor analysis of variance (ANOVA) was performed using SPSS 24 to evaluate significant differences among the treatment groups. Data visualization was conducted using Origin 2024 software to generate various charts illustrating the experimental results. For an in-depth analysis of volatile flavor components, data were processed through the Reporter and Gallery Plot plugins in VOCal (0.4.03), generating two-dimensional chromatograms, difference chromatograms, fingerprint chromatograms, and a compound qualitative table to display the distribution of volatile substances across samples. Additionally, a three-dimensional principal component analysis (PCA) plot was generated using SIMCA 18 software to visually present the distribution of flavor components among different samples. These analytical methods provided valuable insights into the variations in flavor and component distribution across the samples. All experiments were performed independently at least three times.

## 3. Results and Discussion

This section may be divided by subheadings. It should provide a concise and precise description of the experimental results and their interpretation, as well as the experimental conclusions that can be drawn.

### 3.1. Characterization of MT/P34HB Films

#### 3.1.1. FTIR Analysis of Films

**Figure 1:**
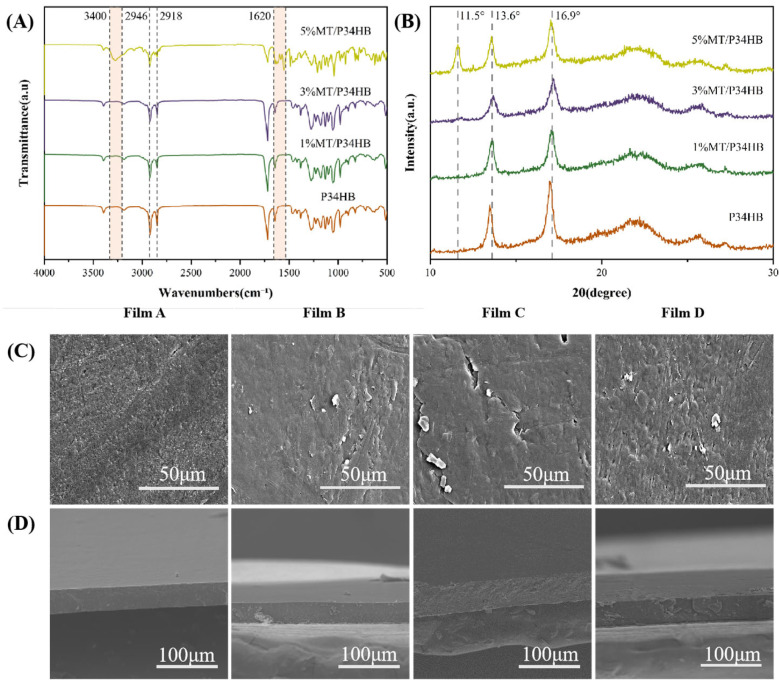
(**A**) FTIR and (**B**) XRD of different packaging films; microstructure of the film’s plane (**C**) and cross-section (**D**).

As shown in Figure 1A, all film groups exhibit C-H bonds characteristic of methyl(–CH₃) and methylene (–CH_2_–) groups, observed at 2918 cm^−1^ and 2946 cm^−1^ [27]. Melatonin contains (-OH) and (N-H) groups, which are likely to form hydrogen bonds with the P34HB matrix [28], gradually reducing the intensity of the characteristic peak in this region. Due to the low melatonin content and the similarity of its peaks to those of the substrate, melatonin’s characteristic peaks are challenging to distinguish when overlapped [29]. In contrast, films containing 5% melatonin exhibit distinct peaks at 3300 cm^−1^ and 1620 cm^−1^, corresponding to the (N-H) groups, and at 3400 cm^−1^ for (-OH) groups. Additional bands in the spectrum arise from the excitation of C–C and C–N stretches in the indole ring, as well as C–H bending vibrations associated with the indole ring, methylene, amide, and methoxy group [28]. These features more clearly confirm the incorporation of melatonin into the material.

#### 3.1.2. XRD Analysis of Films

During the crystallization of P34HB, the flexible 4HB units are excluded from the PHB crystal region, and the amorphous phase does not affect the crystallization type of P34HB. Consequently, the P34HB in this study can be analyzed with reference to PHB data [30].

As shown in Figure 1B, the peak positions remain constant, indicating that the orthogonal structure of P34HB crystals was unaffected by the addition of melatonin. The XRD pattern of PHB typically displays two prominent peaks at 13.6° and 16.9°, corresponding to the (020) and (110) planes, respectively [31].

In melatonin-blended films, the peak intensity is lower than that of the base film. With increasing melatonin content, the characteristic (020) and (110) peaks initially decrease and then increase, likely reflecting the dual effects of melatonin on P34HB’s crystal structure. As a small molecule, melatonin may act as either a plasticizer or disruptor, modifying the molecular mobility of P34HB and influencing its nucleation and crystal growth. Melatonin may enhance P34HB chain mobility, promoting crystallization. However, excessive melatonin could disrupt the ordered arrangement of P34HB chains, reducing crystallinity. Studies have shown that even low concentrations of amide additives can modify P34HB’s crystallization kinetics, promoting or inhibiting crystallization [32].

In the XRD patterns of P34HB films with varying melatonin concentrations, a distinct peak at 11.5° appears in the 5% MT/P34HB sample but is absent at lower concentrations (1% MT and 3% MT) and in neat P34HB. This 11.5° peak is a characteristic of melatonin, indicating that at higher concentrations, melatonin forms crystalline regions within the P34HB matrix. The absence of this peak at lower concentrations (1% MT and 3% MT) may be due to insufficient melatonin to form distinct crystals, leading to amorphous dispersion. In the 5% MT/P34HB sample, the higher melatonin content exceeds the solubility threshold within the P34HB matrix, leading to melatonin’s aggregation and crystalline formation, resulting in the characteristic peaks in the XRD pattern. This behavior aligns with studies on drug–polymer interactions, where the drug’s crystallinity within the polymer matrix depends on its concentration and compatibility with the polymer [33].

#### 3.1.3. SEM Analysis of Films

The micromorphology of the films is shown in Figure 1C,D. As the melatonin increases, the surface topography of the material transitions from rough to smooth. This transition suggests a degree of compatibility between melatonin and P34HB, as increased interfacial interactions result in a smoother and more uniform surface. In particular, this interfacial compatibility appeared to increase with higher melatonin content, leading to changes in the film’s structure.

Unlike films with minimal or no melatonin content (P34HB and 1% MT/P34HB), those with higher melatonin concentrations (3% MT/P34HB and 5% MT/P34HB) display phase separation, forming an island-like structure. This phenomenon typically occurs when two materials exhibit partial compatibility, leading to distinct compositional differences and microscopic phase separation. Previous studies have explored that varying additive concentrations can induce phase separation in polymer films, resulting in microstructures such as island-like formations, consistent with our observations [34].

The XRD data further support these findings, indicating that melatonin addition reduces the crystallinity and grain size of P34HB, contributing to the smoother surface morphology observed.

#### 3.1.4. Comprehensive Properties of Films

As shown in Supplementary Table S2, increasing melatonin content initially decreases and then increases the tensile strength of P34HB film, while elongation at break consistently increases. This phenomenon may be related to the dispersion of melatonin within P34HB, the interactions between molecular chains, and the potential physical cross-linking structures [35]. At low melatonin content (1%), melatonin, acting as a small molecular additive, may insert itself between P34HB molecular chains during melt blending, increasing the distance between chains. This insertion allows the chains to slide and stretch more easily under external forces, reducing tensile strength and increasing elongation at break [36].

As the melatonin content increases further (3% and 5%), additional interactions and physical cross-linking structures may form between melatonin and P34HB, restricting the molecular chain mobility, which raises tensile strength while maintaining high elongation at break. The oxygen barrier property of the films showed an increasing trend, and the OTR decreased to about 92% of the P34HB group. Generally, a higher crystallinity degree results in a more ordered molecular arrangement, increased crystal phases and size, and greater diffusion energy for gas vapor, thus reducing the diffusion coefficient and enhancing resistance to gas [37]. All groups exhibited hydrophobicity, indicated by contact angles greater than 90°. Changes in the water contact angle were similar to those observed in water vapor transmission rate, with 1% and 3% melatonin concentrations showing comparable effects on film hydrophilicity; WVTR and WCA vary by no more than 2%. At lower melatonin concentrations, the material’s surface becomes denser and smoother. In films with high melatonin content, the WOTR of the film increased by about 40%, and the WCA decreased by about 4%. Hydrophilic amide and hydroxyl groups present on melatonin molecules are distributed across the surface and within the film, increasing hydrophilicity and facilitating water adsorption and diffusion. Additionally, as the melatonin concentration rose, the transmission of the film gradually decreased to 95% of the P34HB group, and the haze gradually increased to 156%. It may cause local variations in the refractive index within the film due to the differing optical properties of the melatonin and the polymer. These local refractive index fluctuations lead to light scattering, which impairs the optical transmission and results in higher haze. This effect may be further exacerbated by the formation of microstructures or aggregates of melatonin within the polymer matrix. From an overall perspective, all films demonstrate high transparency, enabling easy observation of packaged food. These analyses align with the XRD, FTIR, and SEM data.

### 3.2. Effects of Different Packaging Films on Peaches

#### 3.2.1. Changes in the Headspace Composition Within the Packaging Bag

**Figure 2:**
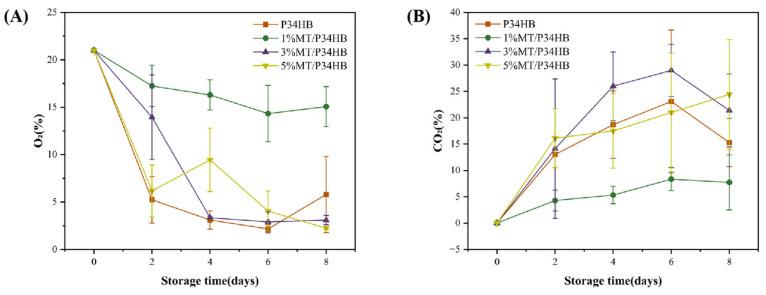
Changes in the concentrations of O_2_ (**A**) and CO_2_ (**B**) within the packaging bag during storage.

As an active packaging material, P34HB film exhibits excellent water barrier properties. It effectively reduces water evaporation through the packaging while allowing a controlled amount of gas exchange, which decreases the respiration rate of peaches and extends their freshness. As shown in Figure 2A, in the 1%MT/P34HB group, the oxygen concentration within the packaging is higher and shows a gradual decline, indicating that an appropriate concentration of melatonin inhibited the aerobic respiration of the peaches. By reducing oxidative damage, melatonin decreased the fruit’s metabolic activity, slowing down oxygen consumption and reducing carbon dioxide production. As shown in Figure 2B, the P34HB, 3%MT/P34HB, and 5%MT/P34HB groups show high carbon dioxide concentration and low oxygen content. These findings indicated that the 1% melatonin concentration was more effective in regulating the microenvironment compared to higher concentrations. At this optimal melatonin level, the film’s antioxidant properties helped maintain a balanced internal gas composition, preserving suitable oxygen and carbon dioxide levels; this phenomenon of melatonin reducing the respiratory rate of peaches is similar to that found in the study by Sati et al. [38]. This balance supported peach respiration in an ideal state, preventing anaerobic respiration, which is typically caused by imbalances in gas concentrations.

#### 3.2.2. Analysis of Basic Physicochemical Properties of Peaches

**Figure 3:**
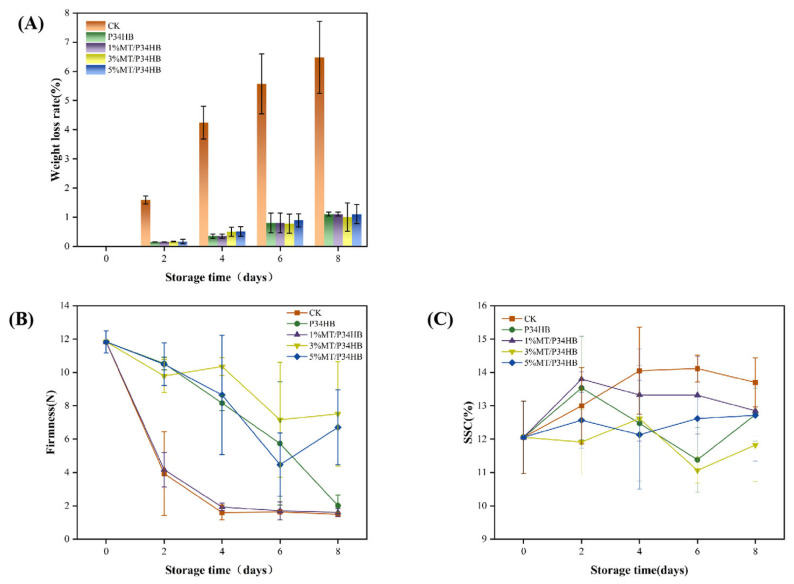
Changes in weight loss rate (**A**), firmness (**B**), and soluble solids content (**C**) of honey peaches during storage.

As shown in Figure 3A, the weight loss rate increased across all groups during the 8-day storage period. The control group (CK) experienced the highest weight loss of 7.8%. In contrast, peaches packaged with P34HB film showed minimal weight loss, with each group losing only about 1% of their weight over the same period. The minor differences in weight loss among the groups treated with different melatonin concentrations suggest that melatonin’s effect on water retention remains relatively consistent across a certain concentration range, without a significant synergistic effect. Additionally, the primary factor controlling the weight loss rate in fruits and vegetables is the barrier properties of the packaging film. It has been reported that proper barrier packaging can effectively control fruit weight loss [39].

As shown in Figure 3B, the firmness of all peach groups exhibited a decreasing trend during storage, a typical characteristic of ripening in melting-type peaches. However, the 3%MT/P34HB and 5%MT/P34HB groups effectively delayed the decline in peach firmness, maintaining a firmness of approximately 6 N at the end of storage, making it three times higher than that of the CK, P34HB, and 1%MT/P34HB. This slower decline in firmness suggests partial inhibition of cell wall degradation. This inhibition likely results from melatonin’s ability to reduce the activity of enzymes involved in cell wall degradation, such as pectinase and polygalacturonase. During postharvest storage, the activity of cell wall-degrading enzymes, including pectinase and polygalacturonase, usually increases gradually, leading to a reduction in fruit firmness and softening. However, melatonin treatment appears to suppress this enzymatic activity [40].

As shown in Figure 3C, the sweetness of peaches is positively correlated with their soluble solid content, a key indicator of fruit sweetness, including sugars, organic acids, and vitamins [41]. In peaches, soluble solids levels directly influence sweetness; generally, the higher the soluble solid content, the sweeter the peach. Peach sweetness depends not only on its inherent composition but also on storage conditions. Under ambient storage, the soluble solid content in the CK group showed an increasing trend, similar to observations from previous studies. In contrast, the soluble solids in the 1% MT/P34HB group were slightly lower than those in the CK group, indicating relatively higher sugar content in the latter. For P34HB, 3% MT/P34HB, and 5% MT/P34HB groups, anaerobic respiration caused a decrease in soluble solids. This is because, during anaerobic respiration, sugars and other soluble substances are converted not into energy but into alcohol or lactic acid, leading to a reduction in total soluble solids. This SSC phenomenon affected by CO_2_ concentration is similar to that reported by Wang et al. [42].

In summary, MT/P34HB film plays a vital role in preserving peach freshness, primarily by reducing water loss and delaying fruit aging to maintain weight and quality. Unlike other groups, peaches packaged in 1% MT/P34HB not only exhibited reduced water loss over the 8-day storage period but also developed a soft texture and sweetness similar to naturally ripened peaches.

#### 3.2.3. Analysis of Volatile Organic Compounds (VOCs) in Peaches

Gas chromatography–ion mobility spectrometry (GC-IMS) is an emerging analytical technology in food science, offering several advantages, including high analytical sensitivity, efficiency, ease of operation, and a simple sample preparation process. This technique has been effectively applied to the analysis of various food types, including fruits [43]. To investigate the effects of different packaging treatments on the volatile organic compounds (VOCs) in peaches, we conducted a comprehensive analysis of the GC-IMS chromatograms for all experimental groups.

##### Characterization of Volatile Compounds in Peaches

The volatile components in all peach samples were qualitatively analyzed, as shown in Supplementary Table S2. A total of 71 signal peaks were detected using the latest NIST and IMS databases, resulting in the identification of 30 volatile flavor compounds, including 8 aldehydes, 5 alcohols, 4 ketones, 12 esters, 1 pyrazine, 1 olefin, and 1 furan compound. Due to database limitations, 21 VOCs could not be identified. In the table, “M” and “D” are used to distinguish between monomers and dimers of the detected VOCs.

##### Analysis of Volatile Compounds During the Natural Ripening of Peaches

In the two-dimensional GC-IMS spectrum topography of VOCs in peaches, the X and Y axes represent ion drift time and retention time, respectively. The shape, size, and color intensity of each spot correlate with the signal peak intensity, indicating the concentration of specific volatile compounds.

**Figure 4:**
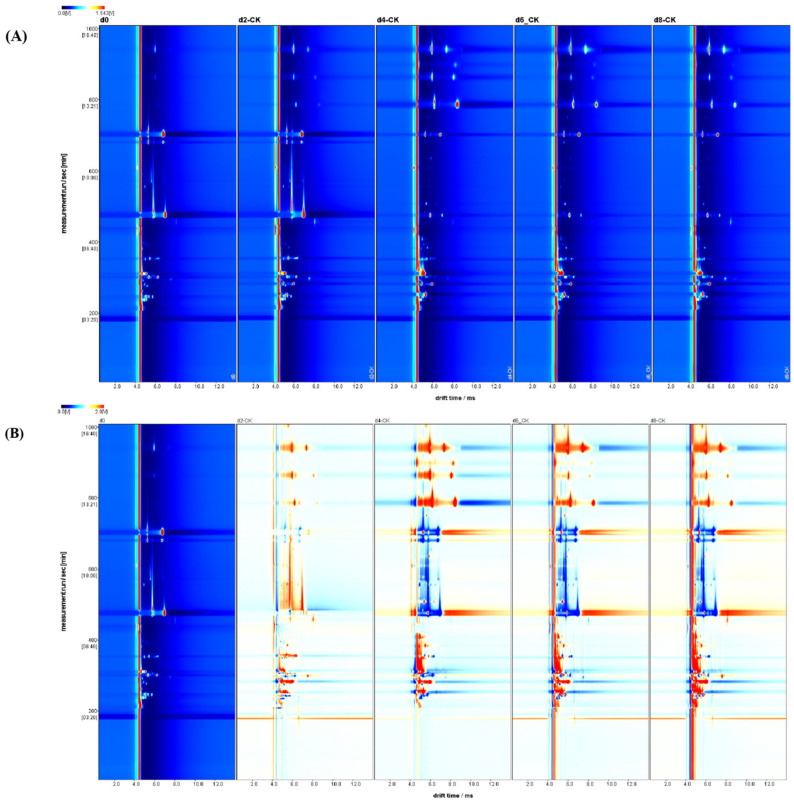
Changes in flavor compounds of naturally ripened honey peaches in the exposed group during storage: 2D plot (**A**) and difference plot (**B**).

As shown in Figure 4A, at d0, freshly picked peaches primarily contained aldehydes, including trans-2-hexenal, 2-hexenal, 3-methylbutanal, and butanal, as well as 2-pentyl furan and 2-isopropyl-3-methoxy pyrazine [44]. These compounds predominantly contribute grassy notes with hints of fruit aroma. Additionally, small amounts of esters, including methyl acetate and sec-butyl acetate, contribute sweet, fruity notes, creating the fresh aroma characteristic of just-picked peaches. This observation was similar to the result reported in a previous study [45]. As shown in Figure 4B, the VOC profile of the CK group was analyzed over 0–8 days of storage under natural conditions, without specific packaging, to examine changes in peach aromatic compounds. If the VOC concentration remains unchanged, it appears in white. Red indicates a higher concentration in the sample than at d0, while blue indicates a lower concentration than the control. During natural storage, we observed that key flavor compounds such as trans-2-hexenal, 2-hexenal, and sec-butyl acetate gradually decreased, while acetic acid ethyl ester, methyl acetate, hexyl acetate, 5-nonanone, (E)-2-octenal, 3-methylbutanal, 1-hexanol, and ethanol increased. Notably, 5-nonanone contributes a woody tone with floral nuances, while trace amounts of ethanol impart an alcohol-like odor. The accumulation of ester compounds, which intensify fruity aromas, shifts the peach’s aroma from a grassy note to a sweeter and more complex fruit profile, giving the characteristic fragrance of ripened fruit [46].

This step in the natural aroma evolution of peaches provides a benchmark for evaluating the impact of different packaging conditions on the aromatic profile of peaches.

##### Changes in the Aroma Characteristics of Peaches Across Different Packaging Groups

**Figure 5:**
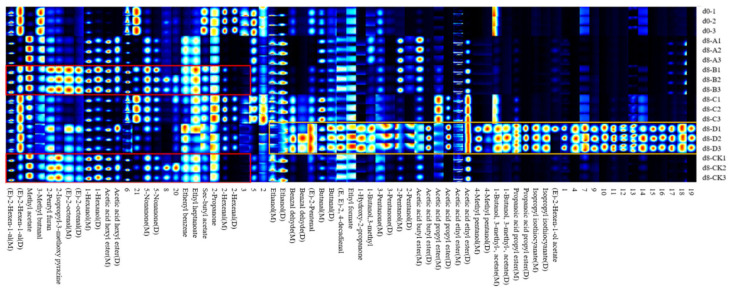
Fingerprint analysis of volatile compounds in peach samples stored for 8 days across different packaging groups, compared to freshly harvested (d0) samples: (d8-A) P34HB, (d8-B) 1% MT/P34HB, (d8-C) 3% MT/P34HB, (d8-D) 5% MT/P34HB and (d8-CK) Unpackaged peaches.

The volatile organic compounds (VOCs) of peach samples packaged with different preservation methods were characterized and analyzed by comparing their IMS retention indices and drift times to those of the control. To examine the effects of different packaging methods on the flavor compounds of peaches during storage, the baseline spectrum of freshly picked samples (group d0) was compared with that of samples stored for 8 days (Figure 5).

In the 5% MT/P34HB group, analysis of the selected areas in the orange box shows that, unlike other groups, many new aroma compounds were formed, including esters, alcohols, ketones, aldehydes, and some unidentified compounds. Among them, the most notable were ethanol and pungent alcohols such as 4-methylpentanol, 1-butanol, and 3-methyl, alongside isopropyl isothiocyanate and 3-pentanone, both known for their sharp odors. Ethyl acetate concentrations also increased significantly, exhibiting a pronounced upward shift along the Y-axis in the GC-IMS two-dimensional map that obscured the signals of other compounds. Ethyl acetate, known for its low aroma threshold, imparts a sweet, fruity scent at low concentrations, but it becomes pungent at higher concentrations, with detectable chemical characteristics [47].

Unlike 1% MT/P34HB and CK groups, the increase in volatile compounds in the 5% MT/P34HB group did not enhance aroma quality. Instead, it produced a chaotic smell dominated by high concentrations of irritating volatiles. This phenomenon was likely due to excessive anaerobic respiration, converting sugars into alcohols (mainly ethanol), which then reacted with organic acids to form aromatic esters. This ethanol fermentation led to a complex and overpowering odor, with high ethanol content masking other aromas and significantly reducing the fruit’s sensory appeal [48]. In contrast, peaches in P34HB and 3% MT/P34HB groups, after 8 days of storage, exhibited aroma profiles similar to naturally ripened peaches, but with fewer and lower concentrations of aroma compounds than groups 1% MT/P34HB and CK. Furthermore, similar surges in irritating compounds observed in the 5% MT/P34HB group also occurred in P34HB and 3% MT/P34HB groups at 4 and 6 days of storage (Supplementary Figure S1).

For 1% MT/P34HB and CK groups, analysis of the selected areas in the red frame shows that, the ester compound profile of peaches in the 1% MT/P34HB group after 8 days of storage closely resembled that of group CK. Notably, the 1% MT/P34HB group retained more initial aroma compounds found in freshly picked peaches (d0), such as (E)-2-hexen-1-al and sec-butyl acetate. Additionally, key compounds characteristic of peach aroma, such as 3-methylbutanal, and original fruity compounds, including methyl acetate, hexyl acetate, 2-pentyl furan, (E)-2-octenal, 1-hexanol, and 5-nonanone, were observed at higher concentrations. Overall, the 1% MT/P34HB group exhibited a more stable aroma profile throughout the 8-day storage period compared to other packaging groups.

##### PCA-Based Analysis of Volatile Compounds

Principal component analysis (PCA) is a simplified method for analyzing multiple variables, with 3D visualization offering improved accuracy when handling a large set of interrelated original variables [49]. In this study, PCA was established based on the differences in signal intensity of volatile compounds among groups from 0 to 8 days (Supplementary Figure S1). The data from three parallel experiments per each group were averaged to create a single data point, facilitating observation and analysis. The PCA results indicate that the cumulative contribution rate of the three principal components is 70.3%, effectively explaining the correlations among the data of each group.

**Figure 6:**
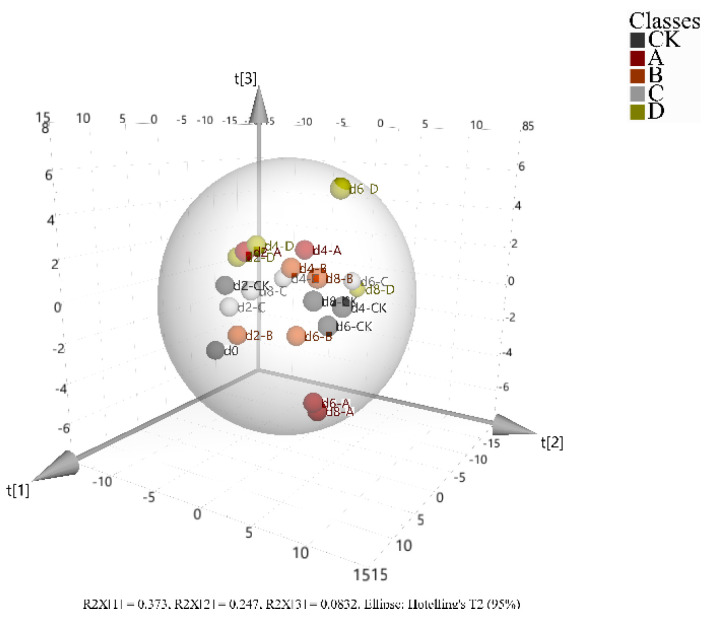
Three-dimensional PCA plot of volatile compound changes in honey peaches: (A) P34HB, (B) 1%MT/P34HB, (C) 3%MT/P34HB, (D) 5%MT/P34HB and (CK) Unpackaged peaches.

As shown in Figure 6, at 0 and 2 days, the peach sample points from different packaging groups were tightly clustered in the same region, indicating that the initial changes in aroma compounds were not substantial enough to differentiate among the packaging types. However, during the 8-day storage period, the sample points of groups P34HB, 3% MT/P34HB, and 5% MT/P34HB exhibited significant dispersion and irregular movement, indicating different changes in peach flavor under varying melatonin concentrations. In contrast, the sample points for groups 1% MT/P34HB and CK remained clustered in the same region throughout the mid-to-late storage periods, with similar movement trajectories. This suggests that the formation and evolution of aroma substances in 1% MT/P34HB-treated peaches resembled those of peaches naturally ripening post-harvest.

#### 3.2.4. Sensory Evaluation

**Figure 7:**
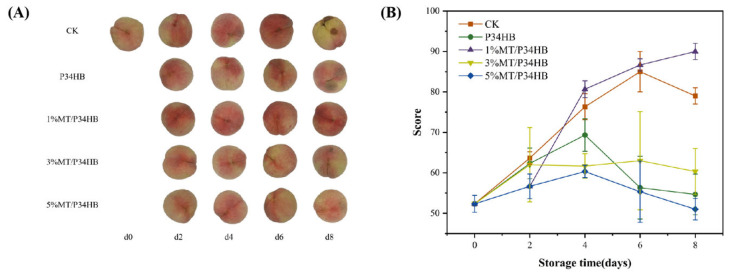
Appearance changes (**A**) and sensory evaluation scores (**B**) of peaches during storage.

As shown in Figure 7A, peaches in the CK group gradually lost moisture at room temperature, with enzymatic browning due to peroxidation at the end of storage [50]. Except for the 1%MT/P34HB group, all other groups covered with the film exhibited slight local darkening on day 8 that was attributed to accelerated decay process from anaerobic respiration. This slight browning did not significantly affect the primary sensory attributes of the peaches, aside from appearance. Figure 7B presents a composite score evaluating appearance, flavor, firmness, juiciness, and sweet–sour balance of the samples. Peaches in the 1% MT/P34HB group maintained their sweetness and a soft texture, similar to those in the CK group, while retaining juiciness better than the CK group. As the 1% MT/P34HB peaches ripened within the packaging, their sensory score steadily increased, reaching approximately 90 points. In contrast, the exposed CK group experienced moisture loss and slight mold growth, leading to a slight decline in sensory score after storage. A similar phenomenon was observed in P34HB, 3% MT/P34HB, and 5% MT/P34HB groups, where ethanol production from excessive anaerobic respiration negatively impacted the taste and flavor of the peaches, resulting in scores between 50 and 70 points, and falling short of consumer expectations for soft, juicy, and flavorful peaches.

## 4. Conclusions

This study demonstrates the potential of melatonin-incorporated P34HB films as an effective active packaging material for regulating the flavor change in honey peaches during storage. Comprehensive evaluations revealed that P34HB films with 1% melatonin optimally preserved the flavor, juiciness, and overall quality of peaches stored at room temperature. Combined with the barrier properties of the film, the melatonin played a crucial role in maintaining a balanced internal atmosphere by effectively controlling the oxygen and carbon dioxide levels and the passage of water within the packaging, thereby minimizing anaerobic respiration and preventing the development of off-flavors. Additionally, the gradual increase in melatonin concentration correlated with a smoother surface morphology of the films, indicating improved interfacial compatibility between melatonin and the P34HB matrix. In practical application, the addition of melatonin further reduces the brittleness of the film and basically maintains the transparency of the film. The findings indicate that the 1% melatonin composite film provided a well-balanced solution, ensuring structural integrity and sensory quality of the peach, making it a promising candidate for fresh-fruit packaging. Further research is encouraged to explore the underlying mechanisms of melatonin’s impact on flavor preservation across various perishable fruits.

## Data Availability

Data are contained within the article.

## Supplementary Materials

The following supporting information can be downloaded at https://www.mdpi.com/article/10.3390/foods14050869/s1. Table S1. Peach sensory scoring rules. Table S2. Physical, Optical, barrier, properties of the MT/P34HB films. Table S3. Characterization of volatile compounds in peaches. Figure S1. Fingerprint analysis of volatile compounds in peach samples stored for 8 days across different packaging groups. Note: (d0) Initial peach sample; (d2/4/6/8-A) P34HB; (d2/4/6/8-B)1%MT/P34HB; (d2/4/6/8-C)3%MT/P34HB; (d2/4/6/8-D)5%MT/P34HB; (d2/4/6/8-CK) unpackaged peaches.

## References

[1] Aubert C., Chalot G. (2020). Physicochemical Characteristics, Vitamin C, and Polyphenolic Composition of Four European Commercial Blood-Flesh Peach Cultivars (*Prunus persica* L. Batsch). J. Food Compos. Anal..

[2] Li X., Gao P., Zhang C., Xiao X., Chen C., Song F. (2023). Aroma of Peach Fruit: A Review on Aroma Volatile Compounds and Underlying Regulatory Mechanisms. Int. J. Food Sci. Technol..

[3] Akbudak N., Eris A. (2004). Physical and Chemical Changes in Peaches and Nectarines during the Modified Atmosphere Storage. Food Control.

[4] Shin J.S., Park H.S., Lee K.W., Song J.S., Han H.Y., Kim H.W., Cho T.J. (2023). Advances in the Strategic Approaches of Pre- and Post-Harvest Treatment Technologies for Peach Fruits (*Prunus persica*). Horticulturae.

[5] Fernández-Trujillo J.P., Salmerón M.C., Artés F. (1997). Effect of Intermittent Warming and Modified Atmosphere Packaging on Fungal Growth in Peaches. Plant Dis..

[6] Cao Z., Zhou D., Ge X., Luo Y., Su J. (2022). The Role of Essential Oils in Maintaining the Postharvest Quality and Preservation of Peach and Other Fruits. J. Food Biochem..

[7] Lin H.-J., Lin Y.-L., Huang B.-B., Lin Y.-T., Li H.-K., Lu W.-J., Lin T.-C., Tsui Y.-C., Lin H.-T.V. (2022). Solid- and Vapour-Phase Antifungal Activities of Six Essential Oils and Their Applications in Postharvest Fungal Control of Peach (*Prunus Persica* L. Batsch). LWT.

[8] Alonso-Salinas R., Acosta-Motos J.R., Núñez-Delicado E., Gabaldón J.A., López-Miranda S. (2022). Combined Effect of Potassium Permanganate and Ultraviolet Light as Ethylene Scavengers on Post-Harvest Quality of Peach at Optimal and Stressful Temperatures. Agronomy.

[9] Yang C., Chen T., Shen B., Sun S., Song H., Chen D., Xi W. (2019). Citric Acid Treatment Reduces Decay and Maintains the Postharvest Quality of Peach (*Prunus Persica* L.) Fruit. Food Sci. Nutr..

[10] Seth T., Asija S., Khatoon S., Iqbal N., Umar S., Khan M.I.R. (2023). A New Perspective of Melatonin in Stress Tolerance through Regulation of Nutrients. S. Afr. J. Bot..

[11] Feng B.-S., Kang D.-C., Sun J., Leng P., Liu L.-X., Wang L., Ma C., Liu Y.-G. (2022). Research on Melatonin in Fruits and Vegetables and the Mechanism of Exogenous Melatonin on Postharvest Preservation. Food Biosci..

[12] Gao H., Zhang Z.K., Chai H.K., Cheng N., Yang Y., Wang D.N., Yang T., Cao W. (2016). Melatonin Treatment Delays Postharvest Senescence and Regulates Reactive Oxygen Species Metabolism in Peach Fruit. Postharvest Biol. Technol..

[13] Wang M., Xu J., Li L., Shen H., Ding Z., Xie J. (2024). Development of Packaging Films Based on UiO-66 MOF Loaded Melatonin with Antioxidation Functions for Spinach Preservation. Food Chem..

[14] Revutskaya N., Polishchuk E., Kozyrev I., Fedulova L., Krylova V., Pchelkina V., Gustova T., Vasilevskaya E., Karabanov S., Kibitkina A. (2024). Application of Natural Functional Additives for Improving Bioactivity and Structure of Biopolymer-Based Films for Food Packaging: A Review. Polymers.

[15] Kumari S.V.G., Pakshirajan K., Pugazhenthi G. (2022). Recent Advances and Future Prospects of Cellulose, Starch, Chitosan, Polylactic Acid and Polyhydroxyalkanoates for Sustainable Food Packaging Applications. Int. J. Biol. Macromol..

[16] Plackett D., Siró I., Lagarón J.-M. (2011). 18—Polyhydroxyalkanoates (PHAs) for Food Packaging. Multifunctional and Nanoreinforced Polymers for Food Packaging.

[17] Boyandin A.N., Dvoinina L.M., Sukovatyi A.G., Sukhanova A.A. (2020). Production of Porous Films Based on Biodegradable Polyesters by the Casting Solution Technique Using a Co-Soluble Porogen (Camphor). Polymers.

[18] Briassoulis D., Athanasoulia I.-G., Tserotas P. (2022). PHB/PLA Plasticized by Olive Oil and Carvacrol Solvent-Cast Films with Optimised Ductility and Physical Ageing Stability. Polym. Degrad. Stab..

[19] Park J.J., Choi Y.H., Sim E.J., Lee E., Yoon K.C., Park W.H. (2023). Biodegradable Poly(3-Hydroxybutyrate-Co-4-Hydroxybutyrate) Films Coated with Tannic Acid as an Active Food Packaging Material. Food Packag. Shelf Life.

[20] Yu Y., Li Y., Han C., Xiao L. (2019). Enhancement of the Properties of Biosourced Poly(3-Hydroxybutyrate- Co-4-Hydroxybutyrate) by the Incorporation of Natural Orotic Acid. Int. J. Biol. Macromol..

[21] Kumari S.V.G., Pakshirajan K., Pugazhenthi G. (2023). Facile Fabrication and Characterization of Novel Antimicrobial and Antioxidant Poly (3-Hydroxybutyrate)/Essential Oil Composites for Potential Use in Active Food Packaging Applications. Int. J. Biol. Macromol..

[22] Kumari S.V.G., Pakshirajan K., Pugazhenthi G. (2024). Development and Characterization of Active Poly (3-Hydroxybutyrate) Based Composites with Grapeseed Oil and MgO Nanoparticles for Shelf-Life Extension of White Button Mushrooms (*Agaricus Bisporus*). Int. J. Biol. Macromol..

[23] Zhao M., Zhang Z., Cai H., Wang L., Hu C., Li D., Chen Y., Kang Y., Li L. (2022). Controlled Moisture Permeability of Thermoplastic Starch/Polylactic Acid/Poly Butylene Adipate-*Co*-Terephthalate Film for the Autolysis of Straw Mushroom Volvariella Volvacea. Food Chem..

[24] Chen P., Wang Y., Li J., Chu W. (2021). Synergetic Effect of Fly Ash Cenospheres and Multi-Walled Carbon Nanotubes on Mechanical and Tribological Properties of Epoxy Resin Coatings. J. Appl. Polym. Sci..

[25] Jiang J., Gong L., Dong Q., Kang Y., Osako K., Li L. (2020). Characterization of PLA-P3,4HB Active Film Incorporated with Essential Oil: Application in Peach Preservation. Food Chem..

[26] Cozzolino R., De Giulio B., Petriccione M., Martignetti A., Malorni L., Zampella L., Laurino C., Pellicano M.P. (2020). Comparative Analysis of Volatile Metabolites, Quality and Sensory Attributes of *Actinidia Chinensis* Fruit. Food Chem..

[27] Brand I., Brand I. (2020). Polarization Modulation Infrared Reflection Absorption Spectroscopy: From Theory to Experiment. Application of Polarization Modulation Infrared Reflection Absorption Spectroscopy in Electrochemistry.

[28] Chakrabarty S., DiTucci M.J., Berden G., Oomens J., Williams E.R. (2018). Structural Investigation of the Hormone Melatonin and Its Alkali and Alkaline Earth Metal Complexes in the Gas Phase. J. Am. Soc. Mass Spectrom..

[29] Nagai N., Okada H., Hasegawa T. (2019). Morphology-Sensitive Infrared Absorption Bands of Polymers Derived from Surface Polaritons. AIP Adv..

[30] Wen X., Lu X., Peng Q., Zhu F., Zheng N. (2012). Crystallization Behaviors and Morphology of Biodegradable Poly(3-Hydroxybutyrate-Co-4-Hydroxybutyrate). J. Therm. Anal. Calorim..

[31] Pryadko A.S., Botvin V.V., Mukhortova Y.R., Pariy I., Wagner D.V., Laktionov P.P., Chernonosova V.S., Chelobanov B.P., Chernozem R.V., Surmeneva M.A. (2022). Core-Shell Magnetoactive PHB/Gelatin/Magnetite Composite Electrospun Scaffolds for Biomedical Applications. Polymers.

[32] Li Y., Han C., Li D., Cheng H., Xiao L., Wang B. (2024). Effects of Linear Diamides Derivative Nucleating Agent on the Enhanced Crystallization and Rheological Properties of Biosourced and Biodegradable Poly(3-Hydroxybutyrate-Co-4-Hydroxybutyrate). J. Therm. Anal. Calorim..

[33] Yan Y., Chen J.-M., Lu T.-B. (2014). Thermodynamics and Preliminary Pharmaceutical Characterization of a Melatonin–Pimelic Acid Cocrystal Prepared by a Melt Crystallization Method. CrystEngComm.

[34] Pervin R., Ghosh P., Basavaraj M.G. (2019). Tailoring Pore Distribution in Polymer Films via Evaporation Induced Phase Separation. RSC Adv..

[35] Liang X., Cha D.K., Xie Q. (2024). Properties, Production, and Modification of Polyhydroxyalkanoates. Resour. Conserv. Recycl. Adv..

[36] Zena Y., Tesfaye M., Tumssa Z., Periyasamy S. (2023). Effects of Modified Elastin-Collagen Matrix on the Thermal and Mechanical Properties of Poly (Lactic Acid). Heliyon.

[37] Chen Q., Auras R., Kirkensgaard J.J.K., Uysal-Unalan I. (2023). Modulating Barrier Properties of Stereocomplex Polylactide: The Polymorphism Mechanism and Its Relationship with Rigid Amorphous Fraction. ACS Appl. Mater. Interfaces.

[38] Sati H., Bhardwaj R., Fawole O.A., Pareek S. (2023). Postharvest Melatonin Application Preserves Quality and Imparts Chilling Tolerance in Peaches. J. Food Biochem..

[39] Chen M., Yan X., Cheng M., Zhao P., Wang Y., Zhang R., Wang X., Wang J., Chen M. (2022). Preparation, Characterization and Application of Poly(Lactic Acid)/Corn Starch/Eucalyptus Leaf Essential Oil Microencapsulated Active Bilayer Degradable Film. Int. J. Biol. Macromol..

[40] Liu Y., Xu J., Lu X., Huang M., Yu W., Li C. (2024). The Role of Melatonin in Delaying Senescence and Maintaining Quality in Postharvest Horticultural Products. Plant Biol..

[41] Simkova K., Veberic R., Hudina M., Grohar M.C., Pelacci M., Smrke T., Ivancic T., Cvelbar Weber N., Jakopic J. (2024). Non-Destructive and Destructive Physical Measurements as Indicators of Sugar and Organic Acid Contents in Strawberry Fruit during Ripening. Sci. Hortic..

[42] Wang D., Ma Q., Belwal T., Li D., Li W., Li L., Luo Z. (2020). High Carbon Dioxide Treatment Modulates Sugar Metabolism and Maintains the Quality of Fresh-Cut Pear Fruit. Molecules.

[43] Leng P., Hu H.-W., Cui A.-H., Tang H.-J., Liu Y.-G. (2021). HS-GC-IMS with PCA to Analyze Volatile Flavor Compounds of Honey Peach Packaged with Different Preservation Methods during Storage. LWT.

[44] Kaufmann A., Maier L., Kienberger M. (2024). Solvent Screening for the Extraction of Aromatic Aldehydes. Sep. Purif. Technol..

[45] Song F., Huangfu Z., Han Y., Li H., Wang Z., Jin X., Chen J. (2024). Nitric Oxide Fumigation Can Affect the Metabolism of Volatile Compounds Derived from Analyses of Fatty Acids and Amino Acids in Post-Harvest Flat Peach during Cold Storage. Food Control.

[46] Xiao Z., Hu Y., Niu Y., Zhang J., Yang B. (2024). Five Representative Esters and Aldehydes from Fruits Can Enhance Sweet Perception. LWT.

[47] Liu Q.-R., Lin X.-L., Lu Z.-M., Chai L.-J., Wang S.-T., Shi J.-S., Zhang S.-Y., Shen C.-H., Zhang X.-J., Xu Z.-H. (2024). Influence on the Volatilization of Ethyl Esters: Nonnegligible Role of Long-Chain Fatty Acids on Baijiu Flavor via Intermolecular Interaction. Food Chem..

[48] Guzel-Seydim Z., Seydim A.C., Greene A.K. (2000). Organic Acids and Volatile Flavor Components Evolved During Refrigerated Storage of Kefir. J. Dairy Sci..

[49] Yang J., Lu R., Tao W., Cai M., Liu G., Sun X. (2024). MultiURNet for 3D Seismic Fault Attributes Fusion Detection Combined with PCA. J. Appl. Geophys..

[50] Yousaf A.A., Sarfraz K., Ahmed A., Hassan I., Ali H., Mehmood T. (2023). Storage Stability Assessment of Indigenous Guava Fruits (*Psidium Guajava* L.) Cv. “Gola” in Response to γ-Irradiation. J. Food Process. Preserv..

