# Supplementary material for: Melatonin/P34HB Films for Active Packaging: Optimizing Flavor Preservation and Quality of Honey Peaches During Storage
Source: Foods. 2025 Mar 3;14(5):869. doi: 10.3390/foods14050869 (PMC11899634; doi:10.3390/foods14050869)
Supplement: Supplementary file 1 [file foods-14-00869-s001.zip › foods-3488574-supplementary.pdf]

Supporting Information

Melatonin/P34HB Films for Active Packaging: Optimizing Flavor Preservation and Quality of Honey Peaches During Storage

1. Supplementary Table S1 Peach sensory scoring rules

Table S1. Peach sensory scoring rules

Score Range	0-3	4-7	8-11	12-15	16-20
Overall Appearance	Significant color irregularities, not matching varietal characteristics, or severe surface damage affecting edibility.	Noticeable color unevenness or prominent surface damage, but still acceptable.	Slight color unevenness or minor surface damage that does not impact edibility	Mostly uniform color with minor differences; minimal surface damage that does not affect appearance.	Bright, uniform color that fully aligns with varietal characteristics; undamaged surface.
Aroma Evaluation	Little to no aroma or entirely off-putting scent, with no recognizable peach fragrance	Weak aroma with evident off-notes, though peach scent is discernible.	Moderate aroma with slight off-notes, requiring close sniffing to detect.	Noticeable peach aroma, with minor off-notes not impacting overall impression.	Strong, pure peach aroma with no off-notes.
Firmness	No firmness; flesh is mushy and unfit for consumption.	Firmness is notably too hard or too soft, diminishing eating experience.	Firmness noticeably too hard or too soft, impacting enjoyment.	Slightly hard or soft with a moderate crispness.	Slightly firm or soft with desirable tenderness.
Juiciness	Flesh is shriveled and dry, lacking juice.	Flesh is relatively dry with insufficient juice.	Moderately juicy flesh, though juice is limited.	Juicy flesh with a moderate amount of juice.	Abundantly juicy flesh with rich juice content.
Sweetness-to-Acidity Balance	Extreme imbalance between sweetness and acidity, resulting	Clear imbalance between sweetness and acidity, creating a poor taste.	Slight imbalance between sweetness and acidity, yet still acceptable.	Balanced sweetness and acidity, providing good taste.	Perfectly balanced sweetness and acidity, offering excellent taste.

	in very poor taste.

This table describes in detail the scoring criteria for sensory evaluation of peach samples.

2. Supplementary Table S2 Physical, Optical, barrier, properties of the MT/P34HB films

Table S2. Physical, Optical, barrier, properties of the MT/P34HB films.

Films	P34HB	1%MT/P34HB	3%MT/P34HB	5%MT/P34HB
**Physical properties**				
Tensile strength (MPa)	16.03±0.61b	12.74±1.13a	13.39±1.25a	13.79±0.39a
Elongation at break (%)	4.87±0.2a	11.63±2.94c	6.13±0.29ab	8.73±0.76b
**Optical properties**				
Transmission (%)	85.57±0.2c	84.9±0b	85.6±0c	81±0.17a
Haze (%)	49.28±0.06a	62.90±0.02b	63.97±0.08c	76.88±0.75d
**Barrier properties**				
Water vapor transmission rate (g/m2·24h)	103.76±2.66a	116.41±5.49b	102.06+±3.34a	141.01±10.47c
Oxygen transmission rate (cm3 /m2 .24h.0.1MPa)	446.56±5.68d	423.38±2.73c	402.64±3.19a	411.79±7.98b
Water contact angle (°)	98.7±3.05b	97.8±0.76b	100.17±0.76c	93.8±4.33a

Note: a, b, c superscript letters represent significant difference, the same letter is not significant difference (P > 0.05), different letters are significant difference (P < 0.05).

3. Supplementary Table S3 Characterization of volatile compounds in peaches.

Table S3. Characterization of volatile compounds in peaches.

Count	Compound	CAS#	Formula	MW	RI	RT[sec]	DT[a.u.]
**Aldehydes**							
1	(E)-2-Pentenal	C1576870	C5H8O	84.1	1146.3	559.01	1.10333
2	(E)-2-Hexen-1-al(D)	C6728263	C6H10O	98.1	1228.9	702.916	1.51081
3	(E)-2-Hexen-1-al(M)	C6728263	C6H10O	98.1	1226.1	698.811	1.17881
4	(E)-2-Octenal(D)	C2548870	C8H14O	126.2	1425.5	1077.31	1.81977
5	(E)-2-Octenal(M)	C2548870	C8H14O	126.2	1425.5	1077.277	1.34418
6	(E,E)-2,4-Decadienal	C25152845	C10H16O	152.2	1365.3	942.894	1.41245
7	2-Hexenal(D)	C505577	C6H10O	98.1	1214.5	682.133	1.50761

8	2-Hexenal(M)	C505577	C6H10O	98.1	1214.3	681.841	1.18295
9	3-Methylbutanal	C590863	C5H10O	86.1	926	302.383	1.19631
10	Benzaldehyde(D)	C100527	C7H6O	106.1	1541	1391.069	1.4759
11	Benzaldehyde(M)	C100527	C7H6O	106.1	1540.7	1390.138	1.15552
12	Butanal(D)	C123728	C4H8O	72.1	835	247.506	1.28304
13	Butanal(M)	C123728	C4H8O	72.1	838	249.11	1.11047
**Alcohols**							
14	1 -Hexanol(D)	C111273	C6H14O	102.2	1364.9	942.041	1.65172
15	1 -Hexanol(M)	C111273	C6H14O	102.2	1365.8	943.97	1.32828
16	1-Butanol, 3-methyl	C123513	C5H12O	88.1	1217	685.666	1.24715
17	2-Pentanol(D)	C6032297	C5H12O	88.1	1118.1	509.03	1.42103
18	2-Pentanol(M)	C6032297	C5H12O	88.1	1118.2	509.325	1.20871
19	4-Methyl pentanol(D)	C626891	C6H14O	102.2	1290.3	798.916	1.61308
20	4-Methyl pentanol(M)	C626891	C6H14O	102.2	1292.9	803.373	1.32859
21	Ethanol(D)	C64175	C2H6O	46.1	940.3	311.99	1.13211
22	Ethanol(M)	C64175	C2H6O	46.1	943	313.908	1.04746
**Ketones**							
23	1-Hydroxy-2-propanone	C116096	C3H6O2	74.1	1292.2	802.103	1.21367
24	2-Propanone	C67641	C3H6O	58.1	824.4	241.806	1.09831
25	3-Pentanone(D)	C96220	C5H10O	86.1	997.4	353.779	1.3538
26	3-Pentanone(M)	C96220	C5H10O	86.1	998.9	355.188	1.11581
27	5-Nonanone(D)	C502567	C9H18O	142.2	1325.7	863.709	1.81547
28	5-Nonanone(M)	C502567	C9H18O	142.2	1328.1	868.315	1.33762
**Esters**							
29	Ethyl formate	C109944	C3H6O2	74.1	823.1	241.1	1.07474
30	Acetic acid butylester(D)	C123864	C6H12O2	116.2	1025.8	384.648	1.60585
31	Acetic acid butylester(M)	C123864	C6H12O2	116.2	1027.5	386.581	1.23131
32	Acetic acid ethyl ester(D)	C141786	C4H8O2	88.1	896.9	283.61	1.33111
33	Acetic acid ethyl ester(M)	C141786	C4H8O2	88.1	896.7	283.445	1.10074
34	Acetic acid hexyl	C142927	C8H16O2	144.2	1282.5	786.149	1.89365

	ester(D)						
35	Acetic acid hexyl ester(M)	C142927	C8H16O2	144.2	1284.5	789.343	1.38708
36	Acetic acid propyl ester(D)	C109604	C5H10O2	102.1	992.2	349.771	1.47433
37	Acetic acid propyl ester(M)	C109604	C5H10O2	102.1	991.2	349.025	1.16452
38	Ethyl heptanoate	C106309	C9H18O2	158.2	1327	866.319	1.41242
39	1-Butanol, 3-methyl-, acetate(D)	C123922	C7H14O2	130.2	1136.5	541.103	1.73876
40	1-Butanol, 3-methyl-, acetate(M)	C123922	C7H14O2	130.2	1135.8	539.873	1.30409
41	sec-butyl acetate	C105464	C6H12O2	116.2	998.8	355.144	1.21881
42	Propanoic acid propyl ester(D)	C106365	C6H12O2	116.2	1049.3	412.25	1.56816
43	Propanoic acid propyl ester(M)	C106365	C6H12O2	116.2	1049.6	412.648	1.21101
44	(E)-2-Hexen-1-ol acetate	C2497189	C8H14O2	142.2	1344.9	901.17	1.85472
45	Isopropyl isothiocyanate(D)	C2253738	C4H7NS	101.2	1177.5	620.031	1.44365
46	Isopropyl isothiocyanate(M)	C2253738	C4H7NS	101.2	1177.5	619.977	1.19289
47	Methyl acetate	C79209	C3H6O2	74.1	843.2	252.017	1.19711
**Others**							
48	2-Pentyl furan	C3777693	C9H14O	138.2	1261.7	752.707	1.25852
49	2-Isopropyl-3-methoxy pyrazine	C25773404	C8H12N2O	152.2	1396.6	1010.457	1.25046
50	Ethenyl benzene	C100425	C8H8	104.2	1284.3	789.073	1.49048
**Unknown**							
51	1	unidentified	*	0	1292.3	802.301	1.09019
52	2	unidentified	*	0	822.5	240.785	1.14512
53	3	unidentified	*	0	1154.3	574.119	1.24571
54	4	unidentified	*	0	1062.2	428.328	1.25626
55	5	unidentified	*	0	926.1	302.44	1.3915
56	6	unidentified	*	0	1098	476.292	1.29862
57	7	unidentified	*	0	1098.3	476.642	1.38903

58	8	unidentified	*	0	920.2	298.494	1.68026
59	9	unidentified	*	0	1086.4	460.095	1.47371
60	10	unidentified	*	0	978.9	339.674	1.44899
61	11	unidentified	*	0	1028.4	387.638	1.47525
62	12	unidentified	*	0	1049.6	412.629	1.44944
63	13	unidentified	*	0	992.2	349.732	1.41222
64	14	unidentified	*	0	1099.8	479.146	1.4426
65	15	unidentified	*	0	970.9	333.774	1.39842
66	16	unidentified	*	0	1177.4	619.788	1.54805
67	17	unidentified	*	0	1122.6	516.739	1.43046
68	18	unidentified	*	0	1121.8	515.428	1.18552
69	19	unidentified	*	0	1121.9	515.637	1.52234
70	20	unidentified	*	0	1085.6	459.054	1.79806
71	21	unidentified	*	0	1099.1	477.906	1.55414

4. Supplementary Figure S1. Fingerprint analysis of volatile compounds in peach samples stored for 8 days across different packaging groups.

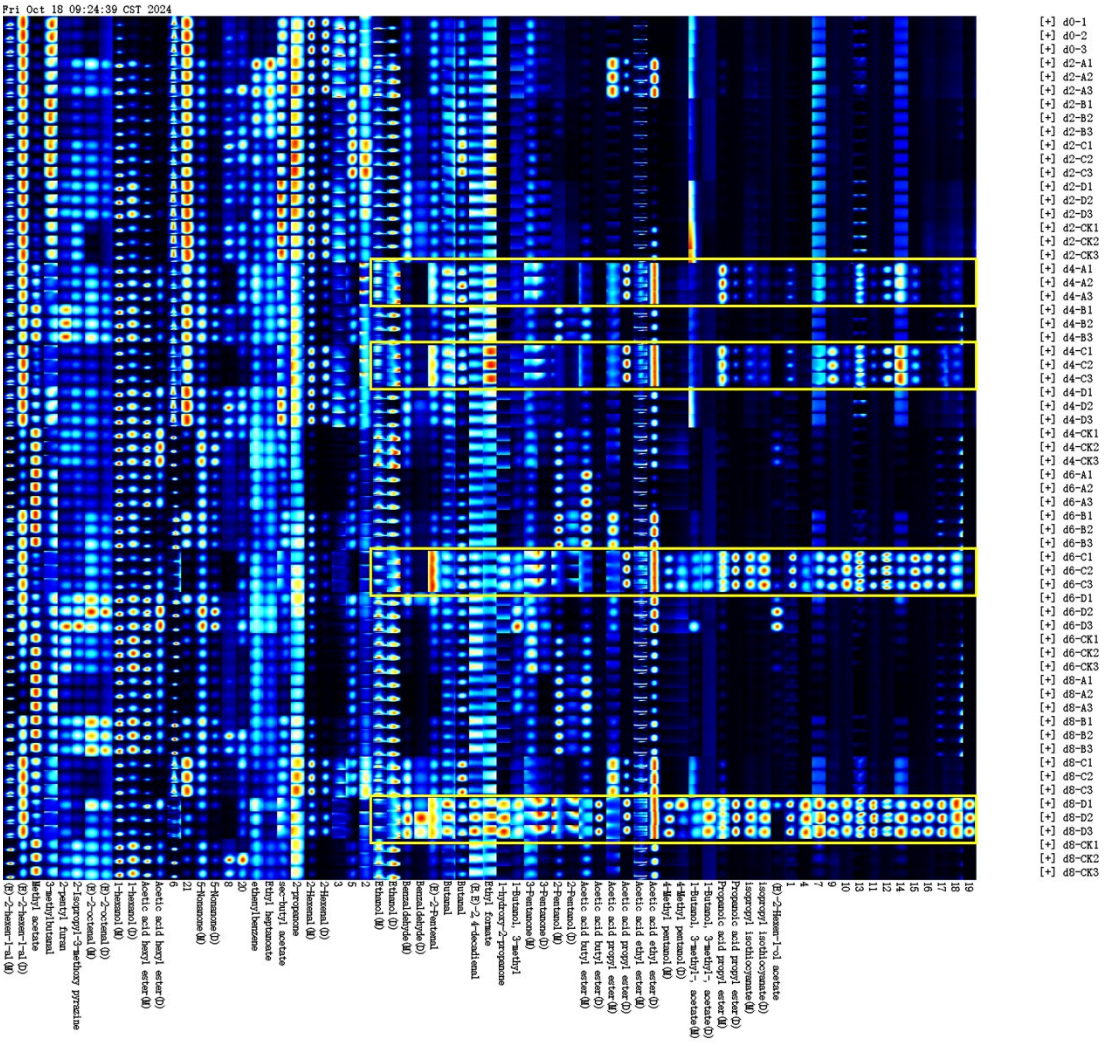

Figure S1. Fingerprint analysis of volatile compounds in peach samples stored for 8 days across different packaging groups. Note: (d0) Initial peach sample; (d2/4/6/8-A)P34HB; (d2/4/6/8-B)1%MT/P34HB; (d2/4/6/8-C)3%MT/P34HB; (d2/4/6/8-D)5%MT/P34HB; (d2/4/6/8-CK) unpackaged peaches.

As shown in Figure_S1, the selected areas in the yellow box are used to support the statement at 3.2.3.3 Changes in the aroma characteristics of peaches across different packaging groups "similar surges in irritating compounds observed in the 5%MT/P34HB group also occurred in P34HB and 3%MT/P34HB groups at 4 and 6 days of storage". It is also used as a reference for the data source of 3.2.3.4 PCA-based analysis of volatile compounds.
